# Patency rate and complications of polytetrafluoroethylene grafts compared with polyurethane grafts for hemodialysis access

**DOI:** 10.3109/03009731003678562

**Published:** 2010-10-27

**Authors:** Hassan Ravari, Gholam Hossein Kazemzade, Mohammad Hadi Saied Modaghegh, Patricia Khashayar

**Affiliations:** ^1^Sina Hospital, Tehran University of Medical SciencesIran; ^2^Mashhad University of Medical SciencesIran; ^3^Research and Development Center, Sina Hospital, Medical Sciences/University of TehranIran

**Keywords:** Hemodialysis, polytetrafluoroethylene (PTFE), polyurethane vascular access graft (PVAG), vascular graft

## Abstract

**Background:**

The survival of hemodialysis patients requiring dialysis depends on the long-term functioning and patency of the vascular access. Prosthetic vascular grafts are inevitably used for patients whose vessels are unsuitable for an autogenous arteriovenous (AV) fistula. The purpose of this study was to compare the patency rate and associated complications using different types of grafts.

**Methods:**

This prospective study was conducted on patients who did not have an appropriate vein for arteriovenous fistula from January 2004 through July 2006. They were divided into two groups, sex, age, and basic data matched. Polytetrafluoroethylene (PTFE) and polyurethane (PVAG) were the two types of grafts used in this study. The functionality of the graft was assessed immediately 1 day and 2 weeks after operation. The clinical follow-up was performed each 3 months until 24 months.

**Results:**

One-year patency rate was reported to be 64% and 52% in the PTFE and PVAG groups, respectively. There was no significant difference in 1-year (64% versus 52%) and 2-year (49% versus 41%) patency rate of the PTFE and PVAG grafts used as vascular access. There was also no difference between the numbers of complications reported in the two groups.

**Conclusion:**

It could be concluded that either PTFE or PVAG grafts can be used with the same expected outcomes.

## Introduction

The number of patients requiring dialysis for end stage renal disease is increasing rapidly. The prolonged survival of this group of patients depends on the long-term functioning and patency of the vascular access for hemodialysis (1). Prosthetic vascular grafts are inevitably used for patients whose vessels are unsuitable for an autogenous arteriovenous (AV) fistula due to several reasons, especially diabetes mellitus-derived atherosclerosis (2).

The artificial polytetrafluoroethylene (PTFE) graft was first developed in 1969 by Robert W. Gore and in 1976, Campell et al. had the first successful experience on human beings. PTFE grafts have been widely used as artificial grafts in vascular surgery; its expanded form has been reported to have acceptable outcomes (3–6).

Different reports have shown low infection rates, being patent for the short and the long term, and not requiring pre-clotting as the advantages of PTFE over older types (such as Dacron) (3,7). The greater need for temporary catheters in individuals receiving PTFEs, however, increases not only the costs but also the risk of infection, bleeding, and poor blood flow (8).

On the other hand, neointimal hyperplasia and distal arteriosclerosis are the most important complications following the administration of these types of grafts (9,10).

Attempts to discover a complication-free graft has led to the invention of the polyurethane vascular access graft (PVAG) (11). Several reports have demonstrated self-sealing properties, low risk of neointimal hyperplasia and coagulability using this graft. Moreover, they can be cannulated within 2 days of placement, avoiding the need for temporary catheters (12). The purpose of this study was to compare the patency rate and associated complications using different types of grafts.

## Material and method

The study was performed as a prospective study from January 2004 through July 2006. Fifty patients in need of a vascular access for the first time, who did not have an appropriate vein for arteriovenous fistula, were enrolled in the study. Cases which developed thrombosis, infection, and other complications, such as venous hypertension and steal syndrome, in which removing the graft was obligatory, were classified as failure.

They were divided into two groups, sex, age, and basic data matched based on the randomized allocation software. A total of 12 females and 13 males participated in each group. The location of the vascular graft, the technique of the surgery, the length of the graft, the stitch string used, the length of arteriovenous anastomosis, and the procedures carried out pre- and post-operation were the same in the two groups. Two surgeons performed an equal number of operations in each group. The patients received 1 g cephazolin intravenous (IV) half an hour prior to the operation as prophylaxis. All the grafts were located on the non-dominant arm. Grafts were washed with normal saline solution and 10,000 units of heparin. Grafts were located subcutaneously so that the arterial anastomosis to the brachial artery and the venous anastomosis to the axillary vein would be near the elbow. End-to-side 8 mm arterial and 12 mm venous anastomosis was performed with prolene 6.0.

Two types of grafts, all 20 cm in length and 8 mm in diameter, were used during this study: 1) polytetrafluoroethylene (PTFE), standard wall manufactured by Gore Co., Flagstaff, AZ, USA; and 2) polyurethane (PVAG) manufactured by Vasculink Co., Woburn, UK.

The functionality of the graft was assessed immediately 1 day and 2 weeks after the operation. The clinical follow-up was performed each 3 months until 24 months. Examination in each session was carried out via touching the thrill, auscultating the bruit, and assessing the outcome of the performed hemodialysis.

The follow-up was performed for 2 years, and the failed cases and also the survival time of each case were recorded. The subjects who died or underwent transplantation during the study period were censored from the study, and those in need of graft removal due to thrombosis and infection were considered as failure.

The gathered data were entered in SPSS v. 13 and analyzed using Kaplan-Meier and *t* test. Continuous variables were expressed as mean and standard deviation.

## Results

There was no significant difference between the age and the gender of the patients studied in the two groups (Table I). The difference in the numbers of diabetics in the two groups was not statistically significant (Table II).

**Table I.** The demographic data of the studied patients in the two groups.

	PTFE (*n* = 50)	PVAG (*n* = 50)	*P*-value
Sex			
Male	24	29	0.212
Female	26	21	
Age (yrs)	57.64 ± 13.3	61.06 ±12.29	0.185
Previous dialysis duration (yrs)	3.96 ± 1.76	3.90 ± 1.80	0.866
Mean arterial blood pressure (MAP) (mmHg)	4	4.2	0.506
Venous diameter (mm)	6.33	9.4	0.002

**Table II.** The prevalence of the underlying diseases leading to dialysis requirement in the studied patients.

	PTFE (*n* = 50)	PVAG (*n* = 50)
Diabetes mellitus	35	31
Renal diseases	25	26
Hypertension	28	37
Tobacco use	28	34
Obesity	17	19
Other	4	2

The mean arterial diameters in the PTFE and PVAG groups were 4 and 4.2 mm, respectively; the corresponding mean venous diameters were 6.33 and 9.4 mm.

The 1-year patency rates were 64% and 52% in the PTFE and PVAG groups, respectively. The 2-year patency rates were 49% and 41%, correspondingly. The mean survival rates of the patients receiving PTFE and PVAG in the same time period were 16 ± 2 (ranging between 13 and 20) and 14 ± 2 (ranging between 10 and 18) months. No significant difference was reported in these groups (*P*-value = 0.35).

From the 50 patients enrolled in the study, 4 (8%) expired, and 2 (4%) underwent transplantation during the study period. Table III shows the number of censored and failed cases in each group. Failure occurred in 5 and 6 cases of PTFE and PVAG, respectively. A significant difference was not found between these two groups (*P*-value = 0.77).

**Table III.** The number of censored and failed cases.

	PTFE (%)	PVAG (%)	*P*-value	95% CI
Censored				
Transplant	2 (8)	-	0.038	0.0954–0.31046
Death	2 (8)	2 (8)	1.000	−0.21279–0.21279
Failure				
Thrombosis	3 (12)	5 (20)	1.000	−0.24789–0.24789
Infection	2 (8)	1 (4)	0.646	−0.13374–0.21374

Infection, steal syndrome (diagnosed based on clinical symptoms including pallor, diminished pulse (distal to the fistula), necrosis, decreased wrist-brachial index (ratio of blood pressure measured in the wrist and that of the upper arm), and pain distal to the fistula), and venous hypertension were the most common complications. There was no significant difference between the numbers of cases experiencing complications during the study period in the two groups.

## Discussion

Due to significant increase in the number of patients in need of hemodialysis, the use of vascular grafts in those without a suitable vein for arteriovenous fistula has increased significantly. The long-term survival of these patients, hence, depends on the appropriate function of these vascular accesses.

Nowadays, polytetrafluoroethylene (PTFE) and polyurethane (PVAG) are among the most frequently used vascular grafts in the world (1,6,11). Several studies have reported different complications and patency rates for these artificial grafts. Some studies have shown new PVAG grafts to be better than the PTFE ones in terms of early access and prompt hemostasis; however, others have questioned the long-term patency and safety of PVAG grafts (13–17).

In a study performed in Australia, PVAG was used in 92% of the procedures in which vascular access was required in an emergency setting. According to this study, a problem-free (primary) and functional (secondary) patency was reported in 44.9% and 64.5% of the patients, respectively. It was concluded that PVAG is the graft of choice in patients requiring an urgent, reliable, medium- to long-term hemodialysis access (18). On the contrary, other studies have shown PTFE to be an efficient vascular access for hemodialysis where a primary fistula or brachio-basilic transplantation is not possible (6,17,19). Modarai et al., however, reported a poor patency rate and a high risk of complication (infection and thrombosis) using this graft (20). A study conducted in 2005 showed that the temporary catheters used in PTFE grafts increase not only the costs but also the risk of infection, bleeding, and poor blood flow. It also showed decreased bleeding time, risk of infection, and anemia exacerbation with PVAG grafts (21). Similarly, Nakagawa et al. reported several advantages of PVAG over PTFE. These advantages included prompt hemostasis and sufficient mechanical strength along with the absence of persistent edema, seroma formation, and any change in the elasticity (22). On the other hand, Matsuda et al. experienced few thromboses using PTFE. Primary patency rate was also reported to be significantly better in the group with PTFE; however, there was no considerable difference in the secondary patency rate of the two grafts (19). Glickman et al., conversely, demonstrated the PVAG grafts to have similar patency and efficacy when compared with the PTFE grafts in dialysis patients. They, however, stressed that PVAG can be cannulated early without increasing the infection rate or sacrificing patency (23).

Contrary to the majority of the above-mentioned studies, our prospective study revealed no significant difference in 1- and 2-year patency or in the frequency of the complications between the two grafts, indicating that either PTFE or PVAG grafts can be used with the same expected outcomes in hemodialysis patients in need of a vascular access. Further studies with larger sample sizes, however, are needed to confirm the results of this study.
